# Oral health-related quality of life before and after crown therapy in young patients with amelogenesis imperfecta

**DOI:** 10.1186/s12955-015-0393-3

**Published:** 2015-12-10

**Authors:** Gunilla Pousette Lundgren, Agneta Karsten, Göran Dahllöf

**Affiliations:** Department of Dental Medicine, Division of Pediatric Dentistry, Karolinska Institutet, POB 4064, SE-141 04 Huddinge, Stockholm, Sweden; Department of Pediatric Dentistry, Public Dental Service, Dalarna County, Falun, Sweden; Department of Dental Medicine, Division of Orthodontics, Karolinska Institutet, Stockholm, Sweden

**Keywords:** Enamel, Dental fear, Patient outcomes, Pediatric dentistry, Quality of life, Restorative dentistry

## Abstract

**Background:**

Amelogenesis imperfecta (AI) is a rare, genetically determined defect in enamel mineralization associated with poor esthetics and dental sensitivity. Because the condition is associated with negative social outcomes, this study evaluated oral health-related quality of life (OHRQoL), dental fear, and dental beliefs before and after early prosthetic crown therapy for AI during adolescence.

**Methods:**

The study included 69 patients with AI, aged 6–25 yr: 33 males and 36 females (mean age 14.5 ± 4.3); healthy controls (n = 80), patients with cleft lip and palate (CLP; n = 30), and patients with molar incisor hypomineralization (MIH; n = 39). All matched in age and gender, and all but the CLP group insocioeconomic area. Patients completed three questionnaires measuring OHRQoL (OHIP-14), dental fear (CFSS-DS), and dental beliefs (DBS-R). Twenty-six patients with severe AI between ages 9 and 22 yr received crown therapy and completed the questionnaires twice: before and after therapy.

**Results:**

OHIP-14 scores were significantly higher among patients with AI (7.0 ± 6.7), MIH (6.8 ± 7.6) and CLP (13.6 ± 12.1) than healthy controls (1.4 ± 2.4) (*p* < 0.001). After crown therapy, quality of life problems in the 26 patients with severe AI decreased significantly, from 7.8 ± 6.1 to 3.0 ± 4.8 (*p* < 0.001). Early prosthetic therapy did not increase dental fear or negative attitudes toward dental treatment.

**Conclusions:**

OHRQoL increased after early crown therapy in patients with severe AI. Therapy did not increase dental fear or negative attitudes toward dental treatment.

## Background

**Fig. 1:**
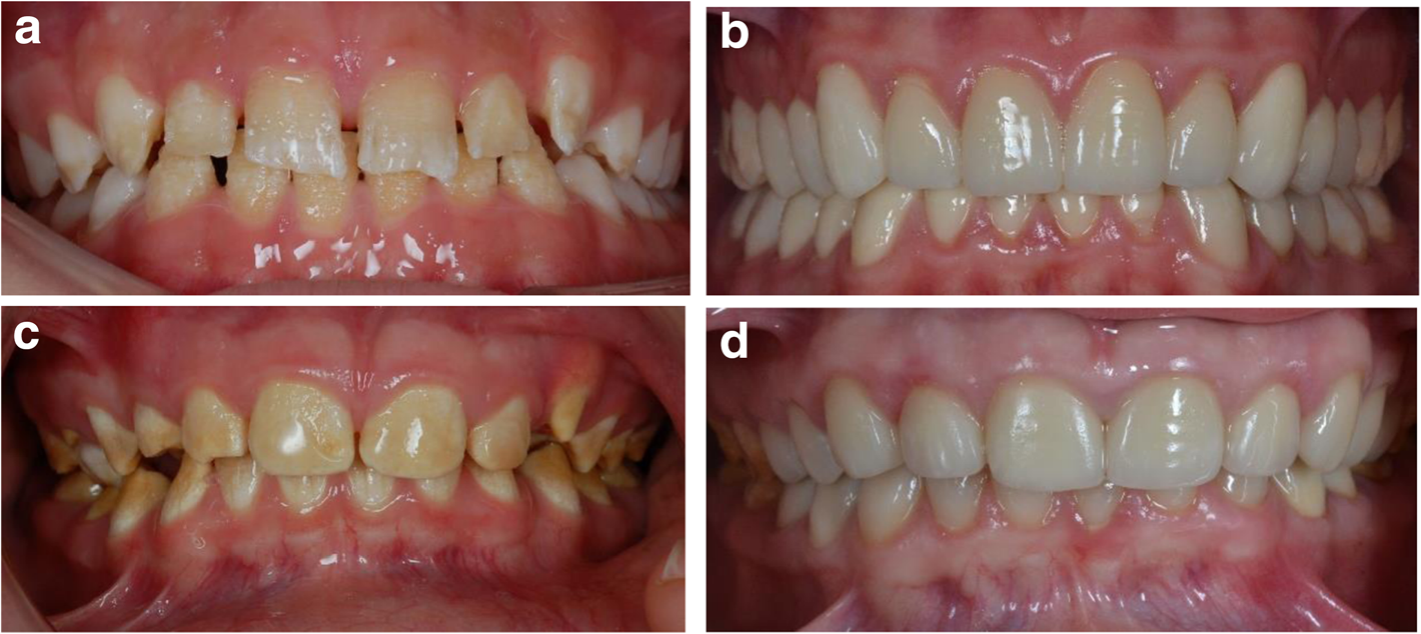
Hypoplastic form of AI before **a** and after **b** crown therapy and hypomineralized/ hypomaturized form of AI before **c** and after **d** crown therapy

Amelogenesis imperfecta (AI) is a rare, genetically determined defect in enamel mineralization with a prevalence between 1:14,000 and 1:700 [1, 2]. Disturbances in the enamel matrix phase result in quantitative defects, as in the hypoplastic form of AI with small, thin teeth, teeth with pits and grooves, or areas without enamel (Fig. 1a before crown therapy, 1 B after crown therapy). Disturbances during the enamel mineralization phase result in qualitative defects, as in the hypomineralized form or hypomaturized form of AI (Fig. 1c before crown therapy, 1 D after crown therapy) [3, 4]. The teeth of patients with AI are prone to disintegration, as well as to hypersensitivity, and patients with AI have more frequent dental appointments and restorations of poorer quality compared to other patients [5]. Current guidelines for restorative treatment suggest covering the surface with direct composite or composite veneers until adulthood and recommend stainless steel crowns for first permanent molars as a temporary solution in childhood and adolescence [6, 7]. Patients with AI often ask for a more permanent treatment at an earlier age [8]. A recent report shows excellent 2-yr survival of porcelain crowns in adolescents and young adults with AI [9].

Oral health-related quality of life (OHRQoL) is the result of an interaction between oral health conditions, and social and contextual factors [10]. AI has major psychological and social implications for OHRQoL. Adult patients with AI report significantly lower OHRQoL compared to controls [11]. Parekh et al. [12] report that children and adolescents with AI have concerns regarding esthetics and function as well as a high level of concern about comments by other people.

Underlying pathological or psychological conditions can also affect patient attitudes toward dentists and dental health care [13]. Young patients with stable favorable dental beliefs had better clinical status and better self-rated oral health than those with less stable beliefs. Factors such as previous experience of pain and low general well-being affect attitudes toward dentistry [14].

The aim of this investigation was to study OHRQoL, dental fear, and anxiety, as well as attitudes towards dentistry in a group of adolescents and young adults with AI. The hypotheses were that adolescents and young adults with AI have lower OHRQoL, higher levels of dental anxiety, and more negative attitudes toward dental care compared with controls; and that OHRQoL in these patients improves after crown therapy.

## Methods

This study followed the Declaration of Helsinki guidelines and was approved by the Regional Ethics Review Board in Uppsala (Daybook number 2008/108).

The study recruited patients from 22 Public Dental Service (PDS) clinics in Dalarna County, Sweden, mainly from rural areas and small towns. PDS clinics in Sweden provide free dental care for children from 3 to 19 years of age and also treat adult patients who pay for their own care. Adults with AI have access to subsidized prosthetic therapy. Study participants were patients with AI who had been referred to the Department of Pediatric Dentistry, PDS in Falun. To be included, patients needed a clinically verified AI diagnosis, confirmed by anamnestic familial history and/or histological examination. We invited 80 patients with AI to participate in this study.

We included three control groups in this study. The first control group was a healthy control group of children with no dental or medical disorders who regularly attended PDS clinics in Dalarna County. The second group was children with molar incisor hypomineralization (MIH); these children exhibit similar problems to patients with AI, including increased sensitivity, frequent dental treatments, and a higher level of dental fear and anxiety [15]. The definition of MIH used in this study was: hypomineralization of systemic origin of one to four first permanent molars that is frequently associated with affected incisors [16]. The third control group comprised patients with cleft lip and palate (CLP); like patients with AI, they exhibit disturbances in dental development, frequent dental treatments, esthetic challenges, and persisting sequelae after completion of therapy. They also report a lower OHRQoL [17, 18].

To form the control groups, we sent invitations to 80 healthy controls and 80 patients with MIH from PDS clinics in Dalarna, and 80 patients with CLP from the Stockholm Craniofacial Center. The three control groups were matched for age and gender to the patients with AI. The healthy controls and the patients with MIH came from the same socioeconomic area as the patients with AI. Patients with AI and healthy controls answered the questionnaires concerning regular dental appointments. All study participants were recruited between December 2008 and February 2013. The parents were asked to assist their children if below nine years of age, explaining the questionnaires and asked to record their children’s experiences. We sent questionnaires to the MIH and CLP groups by land mail in November 2012 and included a self-addressed, stamped envelope. These groups received one reminder 4 months later.

### Questionnaires

We used the 14-item oral health impact profile (OHIP-14) [19], a short version of the OHIP-49 [20], to study OHRQoL. The Swedish version has been tested for reliability and validity [21]. The OHIP-14 is preferable when describing OHRQoL on a population basis and attempting to detect changes over time [22]. It focuses on four dimensions of impact: orofacial pain, oral function, orofacial appearance, and psychosocial impact [23]. The OHIP scales use the five-point Likert scale (never = 0, seldom = 1, sometimes = 2, fairly often = 3, and very often = 4) for responses. We calculated the mean scores for each item and compared them within the groups. Adding the scores for the 14 items gave a total OHIP-14 score ranging from 0 to 56, with higher scores indicating poorer OHRQoL. We excluded subjects with more than five missing OHIP responses from the analyses; if there were five or fewer missing responses, the missing values received the subject’s median response score [23].

The Children’s Fear Survey Schedule – Dental Subscale (CFSS-DS) measures dental fear and anxiety [24]. This psychometric scale consists of 15 items, where each item can give a score from 1 (not afraid) to 5 (very afraid). Thus, possible total scores range from 15 to 75. Calculations suggest a population-based mean value on the CFSS-DS of 23 (SD 8) for 9–11-year-old Swedish children [25]. A score ≥38 indicates dental fear [26]. For the CFSS-DS questionnaires with fewer than five items missing, we replaced missing items using specific-item means [27].

The dental belief survey (DBS-R) examines interpersonal processes and relationships between the patient and the dental care provider [28]. It includes 28 items, covering three dimensions of the interpersonal relationship as conceived by the patient: the ethical dimension, communication, and control. Each item has five score levels from 1 to 5 with 1 indicating no concern and 5 indicating greatest concern. The outcome of the DBS-R is a sum of scores ranging between 28 (highly positive) and 140 (highly negative) [29]. A total score > 42 is the cut-off indicating negative attitudes towards dental care [30]. Patients were excluded if more than 20 % of their items contained missing data [28].

We recorded tooth sensitivity for the entire dentition in patients with AI using a visual analogue scale (VAS), considering a score below 3 to indicate no or low pain [31]

### Measurements before and after early crown therapy in patients with AI

The AI group contained 26 patients diagnosed with severe AI who needed crown therapy. They answered their questionnaires before treatment at their dental examination. After treatment with porcelain crowns (Fig. 1b, d) the patients answered the questionnaires again at the two-year follow-up examination. We calculated change in OHIP-14 score and sub-dimension scores by subtracting the scores after treatment from the baseline scores, so positive values indicated improvement and negative scores worsening of OHRQoL.

To estimate the minimally important difference, we used both anchor- and distribution-based methods. We defined no or low tooth sensitivity (VAS score < 3) after crown therapy as a positive treatment outcome and used it as an anchor. We then estimated effect size (ES) using Cohen’s techniques as the distribution method [32, 33].

### Statistical analyses

The Mann–Whitney *U*-test examined differences between groups. Logistic regression analyses evaluated the influence of age, gender, visits per year, severity of AI, and VAS score on the dependent variables of OHRQoL, dental fear, and dental beliefs. Cronbach’s alpha calculated the internal consistency reliability of the OHIP-14 scale. Treatment effects of crown therapy in patients with severe AI were compared with the Wilcoxon signed rank test, and ES calculated with Cohen’s *d* based on pooled standard deviations. We considered a treatment effect to be trivial if ES was < 0.20, small if 0.2 ≤ ES < 0.5, moderate if 0.5 ≤ ES <0.8, and large if 0.8 ≤ ES.

All analyses were done using the Statistical Package for the Social Sciences (SPSS, ver. 22; Chicago, IL, USA).

## Results

We invited 80 patients with AI to participate in this study and excluded 7 of these because we could not rule out alternative diagnoses. Another 4 did not answer the questionnaires. The remaining 69 patients with AI included 33 males and 36 females (aged 6–25 yr, mean 14.5 ± 4.3). Of these, 33 had hypoplastic type-AI and 36, hypomineralized/hypomatured-type AI. The healthy controls from PDS clinics included 80 patients, 36 boys and 44 girls (aged 6–25 yr, mean 14.7 ± 4.4). Thirty-nine patients with MIH (17 boys and 22 girls aged 8–23 yr, mean 14.7 ± 4.1), and 30 patients with CLP (12 boys and 18 girls aged 6–25 yr, mean 14.8 ± 4.8) returned the questionnaires.

**Fig. 2:**
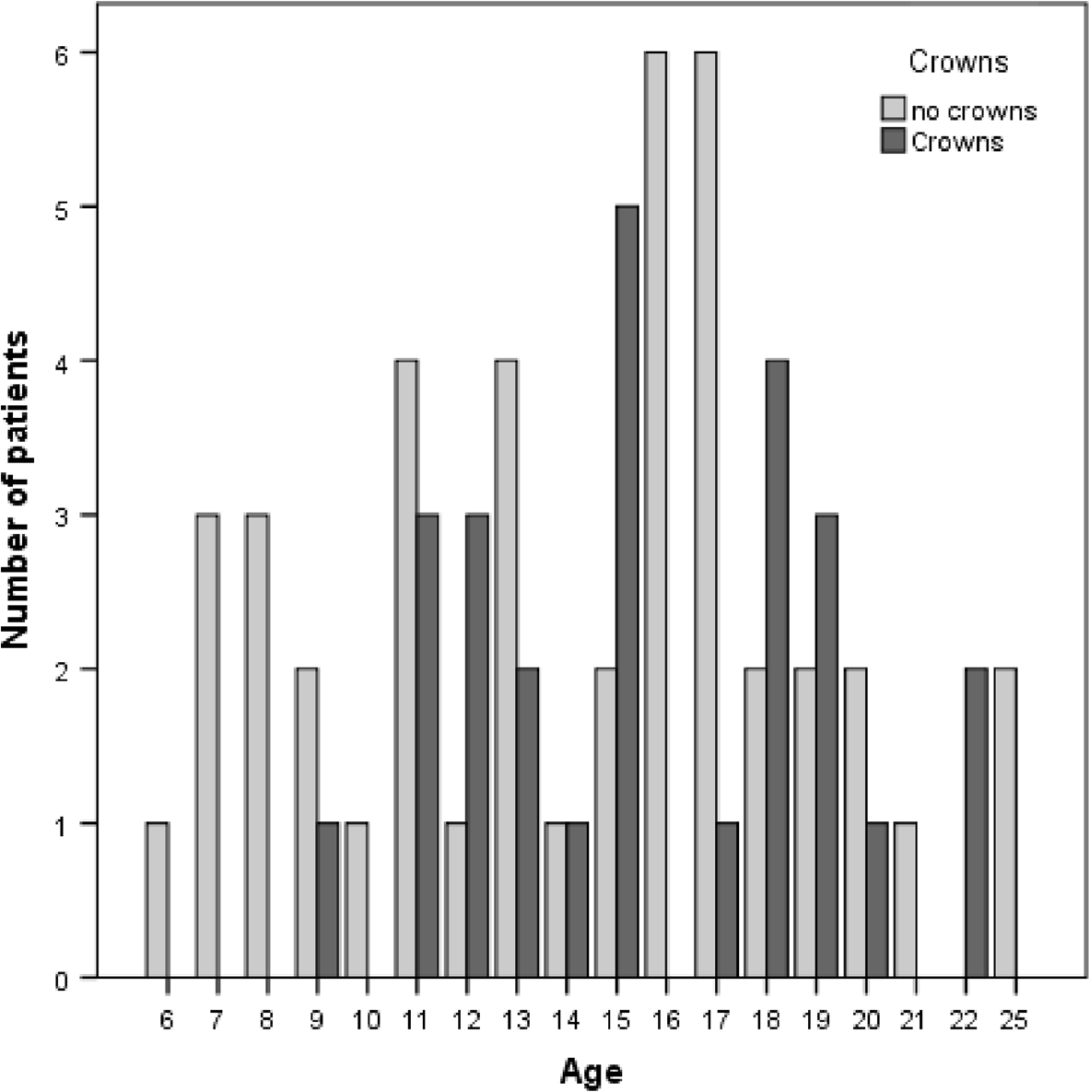
Age of patients with AI in crown group and no crown group

**Fig. 3:**
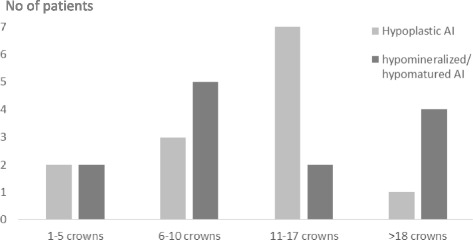
Number of crowns made in each patient and type of AI

Among the patients with AI, 26 (aged 9–22 yr, mean 16.1 ± 3.1) received a diagnosis of severe AI and needed restorative treatment (Fig. 2). The patients received between 1 and 24 crowns each (mean 11 ± 6) (Fig. 3), resulting in 329 crowns total. Four patients receiving crown therapy moved out of the country and did not complete the questionnaires after therapy.

### Quality of life

The total OHIP-14 score was significantly higher among patients with AI (7.0 ± 6.7), MIH (6.8 ± 7.6), and CLP (13.6 ± 12.1) compared to the healthy control group (1.4 ± 2.4). The CLP group scored significantly higher than the AI and MIH groups (Table 1). Within the different OHIP-14 domains, adolescents in the AI group scored significantly higher on all sub-dimensions of the questionnaire compared to the control group (Table 2). Items with the highest score among AI patients were orofacial appearance and orofacial pain. Most problems reported in the control group were in orofacial pain. In the MIH group, orofacial pain and orofacial appearance had the highest scores. Patients with CLP scored high on all four dimensions with orofacial appearance and highest on oral function. Internal reliability (Crohnbach’s α) of the OHIP-14 items in this population aged between 6 and 25 years was 0.886. The individual items varied between 0.869 and 0.892.

**Table 1:** Scores from the Oral Health Impact Profile (OHIP-14), the Children’s Fear Survey Schedule – Dental Subscale (CFSS-DS), and the Dental Belief Survey (DBS-R) in the amelogenesis imperfecta (AI) study group and the three control groups: healthy controls (CTR), molar incisor hypomineralization (MIH), and cleft lip and palate (CLP)

Variables	AI all	CTR	MIH	CLP	Significance^a^
	(*n* = 69)	(*n* = 80)	(*n* = 39)	(*n* = 30)	
	(x ± sd)	(x ± sd)	(x ± sd)	(x ± sd)	
OHIP-14 total sum	7.0 ± 6.7	1.4 ± 2.4	6.8 ± 7.6	13.6 ± 12.1	AI-CTR *p* < 0.001;
					AI-CLP *p* = 0.034;
					MIH-CTR *p* < 0.001;
					CLP-CTR *p* < 0.001;
					MIH-CLP *p* = 0.037
OHIP-14 mean	3.5 ± 3.4	0.7 ± 1.2	3.5 ± 3.8	7.4 ± 7.6	AI-CTR *p* < 0.001;
					AI-CLP *p* = 0.033;
					MIH-CTR *p* < 0.001;
					CLP-CTR *p* < 0.001;
					MIH-CLP *p* = 0.029
CFSS-DS	18.8 ± 5.7	18.8 ± 4.6	24.0 ± 8.9	22.1 ± 8.9	AI-MIH *p* = 0.001;
					AI-CLP *p* = 0.038;
					MIH-CTR *p* < 0.001;
					CLP-CTR *p* = 0.026
DBS-R	32.4 ± 6.4	32.1 ± 5.8	43.3 ± 20.1	38.6 ± 17.2	AI-MIH *p* = 0.002;
					AI-CLP *p* = 0.08;
					MIH-CTR *p* = 0.002;
					CLP-CTR *p* = 0.033

^a^Mann Whitney *U*-test

**Table 2:** Subdimenisons of the Oral Health Impact Profile (OHIP-14) in the amelogenesis imperfecta (AI) study group and the three control groups: healthy controls (CTR), molar incisor hypomineralization (MIH), and cleft lip and palate (CLP)

OHIP-14	AI total	CTR	MIH	CLP	Significance^a^
	(*n* = 69)	(*n* = 80)	(*n* = 39)	(*n* = 30)	
	(x ± sd)	(x ± sd)	(x ± sd)	(x ± sd)	
Oral function	0.29 ± 0.38	0.12 ± 0.24	0.51 ± 0.66	1.14 ± 1.09	AI-CTR *p* = 0.001;
					AI-CLP *p* < 0.001;
					CTR-MIH *p* < 0.001;
					CTR-CLP *p* < 0.001;
					MIH-CLP *p* = 0.013
Orofacial pain	0.61 ± 0.83	0.22 ± 0.48	0.84 ± 1.01	1.13 ± 1.12	AI-CTR *p* = 0.002;
					CTR-MIH *p* < 0.001;
					CTR -CLP *p* < 0.001
Orofacial appearance	1.15 ± 1.31	0.17 ± 0.50	0.70 ± 1.05	1.38 ± 1.31	AI-CTR *p* < 0.001;
					CTR-MIH *p* = 0.001;
					CTR-CLP *p* < 0.001
Psychosocial impact	0.54 ± 0.63	0.06 ± 0.18	0.39 ± 0.52	0.93 ± 1.11	AI-CTR *p* < 0.001;
					AI-CLP *p* = 0.002;
					CTR-MIH *p* < 0.001;
					CTR -CLP *p* < 0.001

^a^Mann–Whitney *U*-test

### Change in OHRQoL after crown therapy in AI patients

**Fig. 4:**
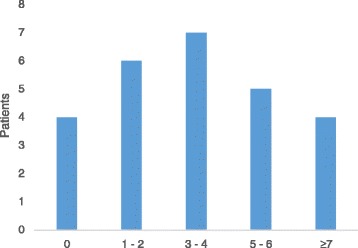
The distribution of improvement in OHIP-14 scores after crown therapy

Two years after crown therapy, OHRQoL improved significantly, total scores decreased from 8.8 ± 5.9 to 2.0 ± 2.5 (*p*< 0.001), and the total mean score for all items decreased from 0.6 ± 0.4 to 0.2 ± 0.2 (*p*< 0.001). Fig. 4 shows the distribution of improvement in OHIP-14 scores after crown therapy. No patient had an increase in total OHIP-14 score after therapy. We found significant improvements in two of four OHIP-14 domains: psychosocial impact and orofacial impact (Table 3).

**Table 3:** Sub-dimension scores of the Oral Health Impact Profile (OHIP-14) in 26 adolescents and young adults with amelogenesis imperfecta before and 2 yr after crown therapy

OHIP-14 dimensions	Sub-score		Significance (p*)	Effect size (95 % CI)
	Before therapy	2 yr after therapy		
	(x ± sd)	(x ± sd)		
Oral function	0.25 ± 0.26	0.18 ± 0.22	0.2180	0.29 (−0.26 – 0.84)
Orofacial pain	0.69 ± 0.84	0.48 ± 0.65	0.2270	0.28 (−0.26 – 0.82)
Orofacial appearance	1.31 ± 1.32	0.28 ± 0.74	0.0014	0.96 (0.26 – 1.66)
Psychosocial impact	0.64 ± 0.64	0.10 ± 0.24	0.0002	1.06 (0.44 – 1.67)
Total score OHIP-14	8.08 ± 5.93	2.04 ± 2.49	0.0001	1.24 (0.62 – 1.85)

* = Wilcoxon signed rank test

The study recorded sensitivity of teeth before and after crown therapy using a VAS scale. Before therapy, 19 of 26 patients (73 %) reported a VAS score > 3 while after crown therapy only three patients (12 %) reported a score > 3.

We calculated the minimally important difference in two ways. The anchor-based method used sensitivity (VAS score < 3) as criteria for an important positive treatment effect. The 19 patients who had a VAS score < 3 after crown therapy had a mean improvement of five points in their OHIP-14 scores. Nine of 26 patients had a decrease of ≥ 5 points in their total OHIP-14 score. For the distribution-based method, estimated ES (Cohen’s d) for the total OHIP-14 score was 1.24 (95 % CI 0.62-1.85) (Table 3).

### Dental fear

The CFSS-DS score in AI patients, 18.8 ± 5.7, was significantly lower than in the MIH group (24.0 ± 8.9, *p* = 0.001) and in the CLP group (22.1 ± 8.9, *p* = 0.038). Differences in CFSS-DS scores between the AI group and the control group (18.8 ± 4.6, Table 1) were non-significant. Among the various items, “injection,” “the dentist drilling,” and “choking” were the most fearful situations for all groups. Total CFSS-DS scores in patients with AI did not differ before and after crown therapy.

### Dental beliefs

Patients with AI scored 32.4 ± 6.4 on the DBS-R, not significantly different from the controls’ 32.1 ± 5.8. Three patients had scores ≥ 42. The highest scores of negative expectations appeared in the MIH group 43.3 ± 20.1, significantly higher than AI patients (*p* < 0.002) and controls (*p* < 0.002), with 15 patients scoring ≥ 42. Regarding specific items, all groups mentioned lack of control as their most troublesome worry. “When I am in the chair, I don’t feel like I can stop the appointment for a rest if I feel the need” was ranked first in the MIH and CLP groups, second in the AI group, and third in the control group.

The total DBS-R score was 31.7 ± 3.9 in the severe AI group before therapy and did not change after therapy (31.2 ± 4.4). In this group, ranking of the item “I am concerned that the dentist is not really looking out for my best interests” fell from the top concern before therapy to twelfth after therapy.

## Discussion

The results of this study showed that adolescents and young adults with AI reported a significantly lower OHRQoL compared to healthy controls and that definitive therapy with porcelain crowns at this age significantly improved OHRQoL. We have also shown that this improvement is clinically significant.

Many dental conditions have psychological and social implications; thus, patient reported outcomes should supplement specific dental outcome measures [34]. In this study, patient age varied between 6 and 25 years. Although the OHIP-14 hasn’t been validated in younger children [35], we decided to use this scale, because the majority were teenagers and young adults. And although the 49 items of the OHIP-49 are reduced to 14 in the OHIP-14, it shows good statistical properties and validity [19].

The determinants of quality of life include biological and physiological factors, symptom and functional status, and the individual’s general health perceptions [36]. For adolescents and young adults, appearance is important. Physical growth combined with cultural pressure to conform to beauty ideals often results in teenagers becoming preoccupied with their own and others’ appearance [37].

AI has consequences for individuals living with the condition. The negative esthetic experience from tooth discoloration and reduced crown size in patients with AI leads to higher levels of social avoidance and distress than subjects without the condition [38]. Patients diagnosed with AI are more often single compared to controls and have fewer children [39].

In this study, children with AI reported a significantly lower OHRQoL compared to healthy controls. This agrees with two previous studies of adult patients with AI [11, 39]. The mean total OHIP-14 score in the study of AI patients with a mean age of 36 years was 25 [39], compared to a mean score of 7 in our group of patients with a mean age of 15. This can be explained by the many years of living with an esthetically suboptimal dentition, increased sensitivity, and frequent dental visits for replacement of restorations [5].

Adolescents and young adults with CLP had a significantly poorer OHRQoL compared to controls, patients with AI, and patients with MIH. A previous study also reported poor OHRQoL in teenagers with CLP [40]. The Child Oral Health Impact Profile score was negatively correlated with depressive symptoms and positively correlated with factors such as self-concept and mastery. The presence of appearance-altering conditions does not necessarily result in poor psychological well-being because social, environmental, and psychological factors may mediate coping and adjustment in patients.

It is important to identify patients or groups of patients at the greatest risk of suffering from a low OHRQoL due to poor oral health (or, as in this case, due to congenital dental aberrations) independently of clinical oral health factors. Reports even suggest that children of mothers who report poor OHRQoL are at greater risk of a low OHRQoL later in life [41]. The low scores on OHRQoL among AI patients is a consequence of the current treatment paradigm. The change we observed after treatment strongly suggests that this paradigm should be altered to include early prosthetic therapy.

Minimally important difference is an important concept when interpreting results from longitudinal studies of the effects of dental treatment on OHRQoL. It is usually defined as the smallest difference in score that patients perceive as beneficial, and which would mandate a change in patient management in the absence of troublesome side effects and costs [42]. The results clearly show a significant positive effect on OHRQoL for crown therapy in patients with AI. The OHIP-14 score reduced significantly compared to controls, and none of the patients reported a worsening score. We have previously shown a significant reduction in tooth sensitivity after crown therapy in patients with AI [9]. A reduction in VAS pain score below 3, which indicates no pain or low pain that doesn’t require analgesics [43], was our standard for determining a clinically important difference in OHRQoL. We found that the OHIP-14 scores of patients who reported a VAS pain score below 3 decreased by 5 points after therapy and that nine patients of 26 (35 %) had a ≥ 5-point reduction. This is similar to the minimally important difference for OHIP-14 reported in a study of adult patients receiving periodontal therapy, which reported a 5-point decrease for a third of the patients [32]. ES calculations are useful when the measurements have no intrinsic meaning (points on a scale) and are independent of sample size [44]. In this study, the ES (Cohen’s *d*) for within-group comparisons was 1.24, which is a very large treatment effect [45]. Parental reports on the Early Childhood Oral Health Impact Scale after treatment under general anesthesia for early childhood caries yielded an even larger ES of 2.1 [46]. For comparison, Mashoto et al. [47] reported an ES of 0.2 after atraumatic restorative treatment in Tanzanian schoolchildren using the Child-Oral Impacts on Daily Performance instrument. None of the patients with AI who received crown therapy reported a worsening OHRQoL, which is an important aspect of their responsiveness to treatment that adds strength to the results [48].

Both patients with MIH and patients with CLP showed higher levels of dental fear. For children with CLP, extensive medical treatment naturally contributes to this. The extensive therapy in the severe AI group, however, did not cause a worsening of dental fear. This might be due to a positive treatment outcome, comparable the increased satisfaction after surgery that patients with CLP experience, resulting in more positive OHRQoL scores [17]. Skaret et al. found a tendency toward increased dental fear in 20-year-old dental patients having more than one medical experience [14]. The patients most severely affected with AI received treatment at the pediatric dentistry clinic while almost all patients with MIH were treated in the PDS clinics. For the patients with AI, treatment approaches sought to introduce treatment in an individualized way and to use analgesics and nitrous oxygen to minimize stress and procedural pain. After receiving their first crowns, patients were asked to decide whether to stop or go on with more therapy.

Young people with stable favorable dental beliefs from adolescence through adulthood have better clinical status and better self-rated oral health than those with less stable beliefs [13]. Patients with AI did not express negative attitudes towards dental treatment, either before or after crown therapy. Both patients with AI and controls scored lower than a comparable group of Swedish patients [30]. It is well known that patients with MIH have tooth sensitivity problems and undergo many dental treatments; although dental fear decreases over time, the most negative attitudes toward dental treatment occurred in this group [49]. Of the concerns that they reported, lack of control and the need to be listened to when reporting pain were highly ranked. Before treatment, the severe AI group scored the item “I am concerned that the dentist is not really looking out for my best interests” as their top concern. After therapy this item fell to rank 12 of 28 items.

A limitation of the current study was the dropouts in the CLP and MIH groups due to patients not returning the questionnaires. Another limitation was the advice to the PDS clinics not to make composite resin restorations before expected crown therapy in patients with AI. Limitations related to questionnaires include that the questions are fixed and thus may not capture the children’s own experiences of their disease. A qualitative approach could possibly increase our understanding of the experiences of having AI and receiving crown therapy early in life. Strengths of this study include that it studied OHRQoL after treatment and that it estimated the clinically meaningful effect.

## Conclusion

In conclusion, we found that patients with AI rated their OHRQoL significantly lower than healthy controls. OHRQoL improved significantly in these patients after crown therapy. Furthermore, the treatment effect was clinically significant. Extensive dental therapy did not increase dental fear or negative attitudes towards dentistry.
